# Aberrant mitochondrial function in ageing and cancer

**DOI:** 10.1007/s10522-019-09853-y

**Published:** 2019-12-04

**Authors:** Julia C. Whitehall, Laura C. Greaves

**Affiliations:** The Medical School, Wellcome Centre for Mitochondrial Research, Institute of Neuroscience, Newcastle University, Newcastle upon Tyne, NE2 4HH UK

**Keywords:** Mitochondria, Ageing, Cancer, Metabolism, mtDNA mutations

## Abstract

Alterations in mitochondrial metabolism have been described as one of the major hallmarks of both ageing cells and cancer. Age is the biggest risk factor for the development of a significant number of cancer types and this therefore raises the question of whether there is a link between age-related mitochondrial dysfunction and the advantageous changes in mitochondrial metabolism prevalent in cancer cells. A common underlying feature of both ageing and cancer cells is the presence of somatic mutations of the mitochondrial genome (mtDNA) which we postulate may drive compensatory alterations in mitochondrial metabolism that are advantageous for tumour growth. In this review, we discuss basic mitochondrial functions, mechanisms of mtDNA mutagenesis and their metabolic consequences, and review the evidence for and against a role for mtDNA mutations in cancer development.

## Mitochondrial function

**Fig. 1:**
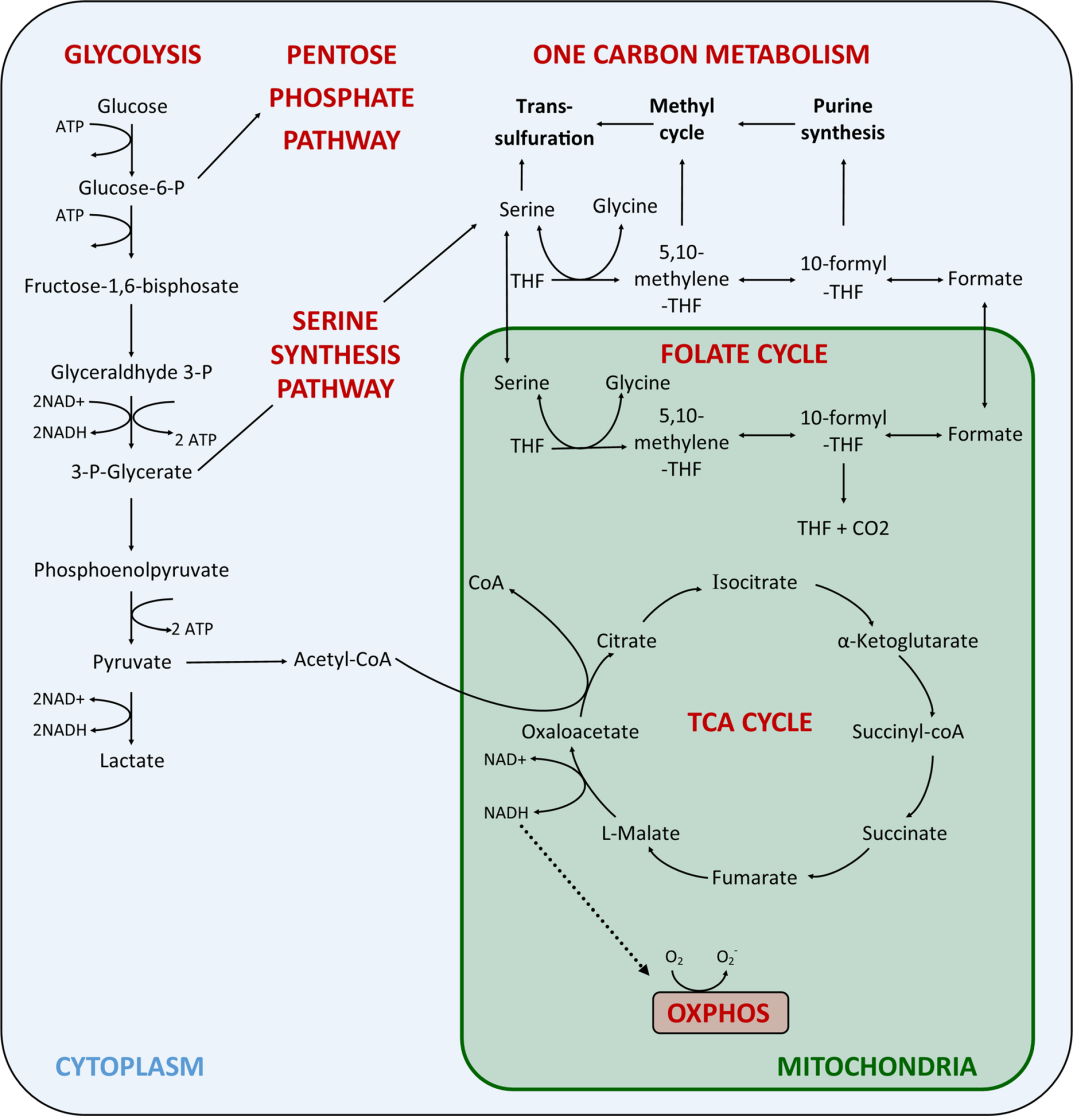
Central carbon metabolic pathways important for cellular energy production and biosynthetic reactions. In the cytoplasm, glycolysis can directly lead to the anaerobic generation of lactate and ATP. Alternatively, in the presence of oxygen acetyl CoA feeds into the mitochondrial TCA cycle to generate electron donors for ATP synthesis via OXPHOS. Depending on the metabolic requirements of the cell, glycolytic intermediates may be diverted into the pentose phosphate and serine synthesis pathways. The SSP produces one-carbon units that supply metabolic pathways such as the mitochondrial and cytoplasmic folate cycles, purine synthesis, transsulfuration and the methyl cycle. *THF* tetrahydrofolate

Mitochondria are present in most eukaryotic cells and are dynamic, intracellular double-membrane bound organelles, which function in a variety of roles to maintain cellular homeostasis. It is thought that the mitochondrion descended from a free-living α-proteobacterium that formed a symbiotic relationship within a host cell (Gray 2012; Gray et al. 1999). During mitochondrial evolution, loss or transfer of the majority of genes from the α-proteobacterium to the nuclear genome has occurred (Karlberg et al. 2000). Subsequently, mitochondria are reliant on the import of nuclear encoded gene products for biogenesis, maintenance and function. However, a small (~ 16 kb) circular genome remains known as mitochondrial DNA (mtDNA), which is essential for one of the major functions of mitochondria; adenosine triphosphate (ATP) synthesis through oxidative phosphorylation (OXPHOS). Other important mitochondrial functions include iron–sulphur biogenesis and haem synthesis (Sano et al. 1959), fatty acid synthesis and oxidation (Hiltunen et al. 2009; Houten and Wanders 2010), one carbon metabolism (Ducker et al. 2016), regulation of reactive oxygen species (ROS), (Diebold and Chandel 2016), calcium homeostasis (Gunter et al. 2000), and induction of apoptosis (Liu et al. 1996). Below we describe in detail some of the normal mitochondrial functions that have been shown to be dysregulated in ageing and cancer (Fig. 1).

## Oxidative phosphorylation

An array of metabolic pathways present within a cell interconnect to convert stored energy from carbohydrates, proteins and fats into energy the cell can utilise in the form of ATP. During glycolysis, glucose molecules enter a series of chemical reactions resulting in the reduction of two nicotinamide adenosine dinucleotide (NAD^+^) molecules to NADH, the generation of two ATP molecules and two pyruvate molecules per glucose molecule. Pyruvate is transported into the mitochondrion where it is decarboxylated to acetyl-coenzyme A (acetyl coA) to allow entry into the tricarboxylic acid (TCA) cycle. One complete turn through the TCA cycle permits the phosphorylation of guanosine-5′triphoshate (GDP), reduction of three NAD^+^ molecules to three NADH molecules and the reduction of one flavin adenine dinucleotide (FAD) to FADH_2_ (Cooper 2000).

NADH and FADH_2_ are both electron donors for energy generation by OXPHOS (Mitchell 1961). In this process electrons are transferred from NADH and FADH_2,_ along the mitochondrial electron transport chain (ETC), which is composed of protein–lipid enzyme complexes (I–IV) located in the inner mitochondrial membrane (IMM), and is coupled via proton pumping to the phosphorylation of adenosine diphosphate (ADP) to ATP by complex V. Electrons entering the ETC at complex I are transferred to ubiquinone (Q), a lipid soluble molecule, which is reduced to ubiquinol (QH_2_) and carries the electrons through the mitochondrial membrane to complex III (cytochrome *c* oxido-reductase). Catalysis of electrons transferred from NADH through complex I to Q, is coupled to the pumping of four protons into the intermembrane space (Hinkle et al. 1991). Electrons donated to complex III from QH_2_, which is re-oxidised to ubiquinone, are transferred from cytochrome *b* in complex III to the haem protein, cytochrome *c,* in the peripheral membrane (Hatefi 1985). The catalysis of 2^e−^ by complex III to cytochrome *c* is coupled to the translocation of 2H^+^ ions into the intermembrane space (Hinkle et al. 1991). Cytochrome *c* carries electrons to the final complex of the ETC complex IV (cytochrome *c* oxidase), which catalyses the irreversible reduction of O_2_, to form H_2_O (Hatefi 1985). Electrons transferred from FADH_2_ to the exclusively nuclear encoded complex II (succinate dehydrogenase), are passed to Q. Complex II (also a participant in the TCA cycle) oxidises succinate to fumarate (Huang and Millar 2013); however, electrons transferred from FADH_2_ to ubiquinone do not release a sufficient amount of free energy for proton pumping. Only free energy yielded at complexes I, III and IV is coupled to the translocation of protons from the matrix across the IMM into the intermembrane space (Hinkle et al. 1991). This sets up an electrochemical gradient across the mitochondrial membrane providing a proton motive force that complex V (ATP synthase) utilizes to phosphorylate ADP to ATP (Hatefi 1985). Movement of protons through the membrane embedded cylindrical F0 domain of complex V, drives the rotation of a central γ subunit (Noji et al. 1997). This rotational motion causes a conformational change of *β* chains in the ATP synthase F1 domain of complex V and the synthesis of ATP from ADP and inorganic phosphate (Noji et al. 1997).

## ROS production

Mitochondria are the major source of cellular ROS production (Loschen et al. 1971), including superoxide, hydrogen peroxide and the hydroxyl radical. Produced by the reduction of O_2_ with unpaired electrons, ROS are highly reactive molecules. A traditional view of ROS centres on their negative role in oxidative stress where they cause damage to cellular proteins, DNA and lipids, and are thought to contribute to disease and ageing (Harman 1956, 1972). Potential sites of endogenous ROS production include enzymes with Flavin-containing prosthetic groups including FAD or Flavin mononucleotide; and enzymes involved in the TCA cycle, ETC and fatty acid oxidation. Within the mitochondria the ETC produces ROS at complexes I and III as by-products during OXPHOS (Sugioka et al. 1988; Turrens and Boveris 1980). Delays during the transfer of electrons down the transport chain facilitates the reaction of stalled electrons with O_2_, producing the free radial superoxide. Furthermore, the strong electrical field produced by the potential difference between the matrix and inter-membrane space can cause a divergence of electrons in the IMM along their path. This electrical field can act as a force, accelerating the movement of superoxide anions formed within the IMM towards the inter-membrane space and thus are more readily accessible to the cytosol (reviewed by Sabharwal and Schumacker 2014).

Prime targets of oxidative damage are therefore mitochondrial enzymes, lipids and mtDNA, which lie in close proximity to the ETC. However mitochondria, do possess their own antioxidant, mitochondrial superoxide dismutase, to scavenge high levels of damaging ROS (McCord and Fridovich 1969; Weisiger and Fridovich 1973). More recently, ROS have been shown to participate in signal transduction oxidation–reduction reactions, primarily oxidising cysteine residues of a range of target proteins to help control important physiological processes (Janssen-Heininger et al. 2008). For example ROS help regulate responses to growth factor stimulation, metabolic excess, low oxygen availability, stresses such as double-stranded DNA breaks, as well as provide signals to regulate autophagy and the inflammatory response (Finkel 2011). Thus, mitochondria play a central role in maintaining homeostasis of ROS levels that are essential for physiological functioning but at high levels can be toxic to a cell (reviewed by (Diebold and Chandel 2016).

## Apoptosis

Apoptosis, also referred to as programmed cell death, is a carefully orchestrated and evolutionarily conserved mechanism for maintaining a healthy cell population, protection against genotoxic stress and pathogen invasion, and the growth and development of an organism (Wang and Youle 2009). Within a cell there are two different apoptotic pathways which are activated by different signals; the intrinsic and the extrinsic pathway. The extrinsic pathway is dependent on cell surface death receptors, whereas the intrinsic pathway is dependent on mitochondrial functioning. Various studies show a crossover and interaction of these two pathways (Li et al. 1998; Luo et al. 1998), with functioning of the mitochondrial dependent apoptotic pathway being essential (Li et al. 2000). Dysregulation of the latter pathway has been shown to lead to abnormal development and disease (Jones et al. 2003; Zhang et al. 2003).

Both pathways involve a cascade of molecular events with cysteine proteases, termed caspases that controllably leads a cell to self-destruct. Controlling this process are a large family of B cell lymphoma (Bcl-2) protein homologs, which comprise anti- and pro-apoptotic proteins (Cory and Adams 2002). Additionally, a further group of heterogeneous proteins, the BH3 family, which act as pro-apoptotic proteins by interacting with and inhibiting the anti-apoptotic Bcl-2 proteins (Youle and Strasser 2008). Focusing on the mitochondrial apoptotic pathway, induction is shown to occur in response to stress including DNA damage, oxidative damage, starvation, serum starvation, chemotherapeutic agents and excess Ca^2+^ (Sattler and Tymianski 2000; Wang and Youle 2009). Such apoptotic stimuli causes Bax and Bak, which under normal conditions shuttle between the cytosol and the outer mitochondrial membrane (OMM), to change conformation and oligomerise in the OMM (Griffiths et al. 1999; Hsu et al. 1997; Wolter et al. 1997). This enables permeabilization of the mitochondrial membrane for release of apoptotic proteins, including cytochrome *c*, which in turn is essential for activating the caspase cascade for programmed cell death to occur (Liu et al. 1996; Lovell et al. 2008; Wang and Youle 2009).

## One carbon metabolism

One-carbon metabolism comprises a network of biosynthetic reactions that occur in the cytoplasm and mitochondria to provide one-carbon units as the building blocks for cellular biosynthesis, methylation and redox reactions (reviewed by Rosenzweig et al. 2018). The one-carbon units are derived from different nutrients, largely the non-essential amino acids serine and glycine (Kalhan and Hanson 2012), which can then flow into the folate cycle and the methionine cycle. Serine and glycine can be sourced exogenously or generated de novo from glucose along a series of enzymatic reactions collectively referred to as the serine synthesis pathway (SSP) (Locasale et al. 2011). The folate cycle can occur in the cytoplasm but occurs more favourably in the mitochondrion (Ducker et al. 2016) with each compartment having their own enzymatic machinery.

The folate cycle first reduces folic acid to tetrahydrofolate (THF), the biologically active form (Newman and Maddocks 2017). THF can then be methylated by serine hydroxymethyltransferase (SHMT) or glycine decarboxylase (GDLC), into methyl-THF in the presence of serine or glycine respectively. Methyl-THF then undergoes a series of redox reactions catalysed by the mitochondrial or cytoplasmic isoforms of methylenetetrahydrofolate dehydrogenase (MTHFD1/2/1L). Notably, a considerable amount of reduced nicotinamide adenine dinucleotide phosphate (NADPH) production, equal to that of the pentose phosphate pathway occurs by the mitochondrial folate pathway (Fan et al. 2014). The generation of formate through this pathway can then feed into purine synthesis or alternatively methyl-THF may enter into the methionine cycle, important for epigenetic modifications, and methylation reactions (Finkelstein 1990) (Fig. 1).

In the methionine cycle methylation of homocysteine by methyl-THF, forms the only essential sulphur-containing amino acid, methionine (Finkelstein 2000). Moreover generation of ATP during the folate cycle can then be used in the methionine cycle to synthesise S-adenosyl-methionine (SAM) by reacting with methionine (Markham et al. 1980); SAM is the major universal methyl-donor in living organisms (Chiang et al. 1996). Amongst a broad range of methylation reactions required for cellular functioning, there is a high demand for mitochondrial 10-formyl-THF which is required for the formylation of the initiator methionine tRNA for protein translation in the mitochondria (Tucker et al. 2011). To complete the cycle homocysteine can also be regenerated by the demethylation of SAM. Of note homocysteine can also branch off into the transsulfuration pathway for the generation of the antioxidant glutathione (Zhou et al. 2017). Thus, mitochondria support one-carbon redistribution essential for purine, ATP, NADP and glutathione synthesis and methylation reactions, which are crucial for controlling cell fate and homeostasis.

## Mitochondrial genetics

The majority of mammalian somatic cells contain many mitochondria. mtDNA copy number is thought to reach 10^3^–10^4^ per cell (Lightowlers et al. 1997), with mature oocytes containing at least 10^5^ copies (Shoubridge and Wai 2007). mtDNA contains 37 genes, which encode 13 protein subunits of the mitochondrial ETC, the mitochondrial translation machinery, in the form of a 12S and 16S ribosomal RNA (rRNA), and a full complement of 22 transfer RNAs (tRNAs). The mitochondrial genome has a unique genetic code (Barrell et al. 1979) with an extremely economical organisation, lacking introns, and only having a few noncoding bases between some genes (Anderson et al. 1981). The only exception to this being the displacement loop (D-loop) region, which contains the three promoter regions of the H- and L-strand (Kasamatsu et al. 1971). Moreover, there is overlap of some protein encoding genes e.g. *ND4* and *ND4L*, and the transcription termination codon for many mRNAs is not encoded but is instead generated by post-transcriptional polyadenylation (Anderson et al. 1981; Ojala et al. 1981).

mtDNA lacks histones and is packaged into nucleoprotein complexes called nucleoids, containing single or multiple mtDNA molecules (Bogenhagen 2012; Satoh and Kuroiwa 1991). The most abundant protein responsible for co-operatively binding and wrapping mtDNA to form these compact segregated units is the HMG-box protein, mitochondrial transcription factor A (TFAM) (Kaufman et al. 2007; Parisi and Clayton 1991). However, other nucleoid core proteins which, like TFAM, also play roles in mtDNA replication and transcription include the mitochondrial DNA polymerase γ (POLγ), mitochondrial RNA polymerase (POLRMT), mitochondrial single-strand binding protein (mtSSB) and the mitochondrial TWINKLE helicase (Bogenhagen et al. 2008). Towards the periphery of nucleoids other proteins involved in translation and complex assembly and those in the IMM e.g. prohibitin, are present, associating the mtDNA with the IMM (Bogenhagen et al. 2008; Falkenberg et al. 2007).

The multi-copy nature of mtDNA means that cells rarely exist in a state where all copies are identical (homoplasmy), in fact, the vast majority of cells contain a mixture of wild-type (WT) and mutated molecules, termed heteroplasmy (Stewart and Chinnery 2015; Taylor and Turnbull 2005). However, the high copy number and highly recessive nature of mtDNA mutations within a cell allows WT mtDNA to compensate for mutated mtDNA up to a certain point (Sciacco et al. 1994). Beyond a critical threshold however, a biochemical defect occurs. The threshold level is dependent on the tissue type, and it’s metabolic demand (Rossignol et al. 1999), as well as the number of residual WT mtDNA copies (Sciacco et al. 1994).

## mtDNA mutations and mitochondrial dysfunction in ageing tissues

The mt.8344A > G transition in the tRNA^Lys^ gene, and mt.3243A > G transition in the tRNA^Leu^ gene, were the first mtDNA point mutations detected to be present at higher levels in homogenate ageing tissues such as brain, heart, liver, kidney and skeletal muscle (Münscher et al. 1993; Zhang et al. 1993). Nekhaeva et al. then used single-cell sequence analysis and showed that a subset of normal ageing buccal epithelial cells and cardiomyocytes contain mtDNA mutations at high levels of heteroplasmy or homoplasmy (Nekhaeva et al. 2002). Since this discovery, an age-related increase in the frequency of cells with high level mtDNA point mutations resulting in OXPHOS defects have been detected in a number of ageing mitotic tissues such as colon, stomach, small intestine, oesophagus and liver (Fellous et al. 2009b; Greaves et al. 2010; McDonald et al. 2008; Taylor et al. 2003).

mtDNA point mutations in ageing tissues appear to occur at random throughout the mitochondrial genome; however, the mechanism underlying their generation has been an area of much debate. The mitochondrial theory of ageing postulates that due to its close proximity to the mitochondrial respiratory chain, the mtDNA is highly susceptible to oxidative damage (Miquel et al. 1980). This can result in base modifications, sugar damage, strand breaks and abasic sites (Wang et al. 1998). Commonly produced base lesions are 7,8-dihydro-8-oxo-2′-deoxyguanoisine (8-oxo-dG) (Bohr 2002) and thymine glycols (Wang et al. 1998). The formation of 8-oxod-G is suggested to induce G:T transversions by POLγ, whereas thymine glycols are thought to be less mutagenic and instead block polymerisation (Alexeyev et al. 2013; Hanes et al. 2006). Analysis of mutational spectra from aged mitotic and post-mitotic human tissues has shown that the pattern is not consistent with the G:C to T:A transversions caused by oxidative damage (Greaves et al. 2014; Kennedy et al. 2013), but instead, the most common mutation type were G:C to A:T transitions. These are more likely caused by errors during mtDNA replication and/or spontaneous cytosine deamination (Zheng et al. 2006). Along with multiple studies of mtDNA mutational spectra in transgenic mouse models (reviewed by Kauppila et al. 2017), these data suggest a limited role for ROS in age-related mtDNA point mutation generation.

Within ageing mitotic cells, mtDNA point mutations are commonly present at high levels of heteroplasmy or homoplasmy. How one mutated mtDNA molecule clonally expands to become the dominant species within a cell, resulting in a biochemical defect, has been debated for a number of years without a definitive mechanism being agreed upon. In 1997 de Grey suggested that individual cells with mutated mtDNA and subsequent respiratory chain deficiency would result in the production of fewer ROS, resulting in lower levels of mitochondrial membrane damage. Mitochondria with WT mtDNA and with normal respiratory chain function would produce more ROS and suffer a higher rate of oxidative membrane damage, and thus more likely be targeted for lysosomal degradation. Mitochondria with mutated mtDNA would therefore have a selective survival advantage and rapidly populate the cell (de Grey 1997). However, this ‘survival of the slowest’ hypothesis (Kowald and Kirkwood 2000), only explains clonal expansion after a respiratory deficiency has been reached and does not acknowledge the dynamic reticular network of mitochondria. Computational simulations have suggested that simple genetic drift is able to explain the clonal expansion of mtDNA mutations in mitotic cell populations (Coller et al. 2001; Stamp et al. 2018). These models suggest that the initial mutational events occur early in life, then through successive cycles of mtDNA replication followed by random segregation at cell division, mutations are either lost or can expand to become the dominant species within the cell, resulting in mitochondrial dysfunction. Additionally, as the stem cells of mitotic tissues are the most-long lived cell types, once a mtDNA mutation becomes fixed within a stem cell, this will be transmitted to all of the stem cell progeny. This results in the patches of cells with OXPHOS defects that have been documented in ageing stem-cell derived populations (Fellous et al. 2009a; McDonald et al. 2008; Stamp et al. 2018).

In addition to the most commonly documented consequences of mtDNA mutations, which are defects in OXPHOS, a number of groups have investigated the downstream consequences of OXPHOS dysfunction on other aspects of mitochondrial metabolism. Nikkanen et al. analysed skeletal muscle from a mouse model of progressive mtDNA mutagenesis caused by a mutation in the TWINKLE gene. They detected an upregulation of the mitochondrial folate cycle and purine synthesis and increased glucose uptake for de novo serine and glutathione synthesis in response to mitochondrial stress (Nikkanen et al. 2016), which was subsequently shown to be controlled via the mechanistic (mammalian) target of rapamycin (mTOR) complex I (mTORC1) pathway (Khan et al. 2017). These pathways were also confirmed to be upregulated in blood and muscle of patients with mitochondrial diseases (Buzkova et al. 2018). Folate-driven one carbon metabolism is the major anabolic biosynthesis pathway, which provides one-carbon units for cell growth and repair. Bao et al. showed that a number of genes in the de novo SSP were regulated at the transcriptional level by the transcription factor ATF4, and ATF4 mediated rewiring of mitochondrial one-carbon metabolism following ethidium bromide mediated depletion of mtDNA in cell lines (Bao et al. 2016). These data show that cells with mitochondrial dysfunction are able to rewire their metabolism in an attempt to maintain cellular homeostasis.

Despite the plethora of evidence showing an association of mtDNA mutations with human ageing, it is still not clear whether these are a cause or consequence of ageing. The mitochondrial mutator mouse model was developed to address this directly. The mutator mouse harbours an error prone mitochondrial polymerase gamma, which causes the accumulation of mtDNA mutations systemically with age (Kujoth et al. 2005; Trifunovic et al. 2004). This results in a premature ageing phenotype with greying of hair, kyphosis, reduced subcutaneous fat, osteoporosis, reduced fertility, anaemia and heart enlargement leading to a reduced lifespan (< 12 months). A number of other groups have used the mutator mouse model to investigate how mitochondrial dysfunction may specifically contribute to ageing processes in specific cell and tissues types. These studies have shown defects in innate immunity regulation (Niu et al. 2007), inflammation (Logan et al. 2014), stem cell activity (Ahlqvist Kati et al. 2012; Chen et al. 2009) and levels of apoptosis (Norddahl Gudmundur et al. 2011). Taken together these studies suggest that, in this model, mitochondrial dysfunction can drive ageing through a combination of perturbations in ROS signalling pathways and defects in cellular bioenergetics (Baines et al. 2014).

## Mitochondrial dysfunction in cancer

### Altered mitochondrial metabolism in cancer cells

Mitochondrial function was first proposed to play a role in cancer biology in the early twentieth century. Cancer cells exhibiting rapid growth and proliferation rates in harsh tumour microenvironments, must adapt their metabolism to meet the high energy demands required for these processes. Warburg first documented tumours undergoing a metabolic switch with increased levels of glucose uptake and a high rate of glycolysis, even in the presence of oxygen (Warburg 1913, 1956a, b; Warburg et al. 1927). This property of tumour cells, termed the ‘Warburg effect’, is still utilised in clinical monitoring of oncology patients by positron-emission tomography (PET), which allows detection of high glucose uptake by primary and metastatic tumours (Di Chiro et al. 1982; Hawkins and Phelps 1988). There is a positive correlation between tumour aggressiveness and glucose uptake (Kunkel et al. 2003). Energy derivation via glycolysis may be seen as a disadvantage, yielding only two ATP molecules per glucose molecule instead of 36 ATP molecules through complete aerobic oxidation. However, switching from mitochondrial OXPHOS to aerobic glycolysis may permit adaptation to the hypoxic tumour microenvironment. ATP generation by glycolysis can occur at a faster rate than OXPHOS (Pfeiffer et al. 2001) and diversion of pyruvate away from OXPHOS to lactate production and anabolic processes such as the pentose phosphate pathway for nucleotide, lipid and amino acid generation, may aid rapid proliferation (Giampazolias and Tait 2016).

Currently there is still much support for the Warburg Effect in tumours. Warburg, however, proposed aerobic glycolysis was in fact due to a defect in mitochondria (Warburg 1956b), suggesting mitochondria were dispensable for the oncogenic process. However, the recognition of mitochondrial outer membrane permeabilisation as a critical point in programmed cell death (Zamzami et al. 1995) and earlier reports of the mitochondrial membrane potential being higher in cancer cells (Chen 1988), implicated the dysfunctional control of mitochondria to be crucial for the evasion of apoptosis by cancer cells (Hanahan and Weinberg 2000). A significant involvement and dependence of mitochondrial functioning in malignant transformation and tumour progression as well as the influence in response to anticancer treatments has become apparent (Porporato et al. 2017). Subsequently, a vast range of studies have appealed for aberrant functioning of mitochondria to be investigated as potential targets for the development of cancer therapies (Fulda et al. 2010; Pustylnikov et al. 2018). Drugs that target mitochondria to re-sensitise cells to apoptosis for example, have been clinically approved for use in treatment e.g. venetoclax for chronic lymphocytic leukemia (Ashkenazi et al. 2017; Sarosiek and Letai 2016).

Metabolic rewiring in tumours is not limited to increases in glucose uptake for ATP synthesis by glycolysis, or upregulation of the pentose phosphate pathway. The harsh and constantly evolving tumour microenvironment requires other metabolic circuits and entries between mitochondrial and cytoplasmic compartments to be suitably co-ordinated and selected for tumour survival. For example, increased glutamine consumption and metabolism feeds into the TCA cycle for ATP synthesis, fatty acid and nucleotide synthesis, is seen in tumours and has been explored as therapeutic channel (Altman et al. 2016). Additionally, cancer cells are shown to recruit and utilise other carbon sources such as acetate, glycine and serine to adapt to and support their growth and proliferation in the tumour microenvironment (Maddocks et al. 2013; Mashimo et al. 2014). The heterogeneity of conditions such as oxygenation, within a tumour has also been shown to set up a variation in metabolic profiles and symbiosis between tumour regions for the exchange of metabolites. Tumour regions under hypoxia utilising glucose for glycolysis and releasing lactic acid, are shown to fuel oxidative tumour cells, thus sparing glucose for neighbouring hypoxic tumour regions (Feron 2009; Sonveaux et al. 2008).

### Nuclear DNA mutations in metabolism genes in cancer

The rewiring and plasticity of mitochondrial metabolism and signalling in aiding tumourigenesis has been highlighted from the gene level up to the protein and functional level. At the genetic level tumours have been reported to harbour mutations in nuclear encoded enzymes involved in metabolism and accumulate associated oncometabolites, thought to increase tumorigenesis. In numerous types of tumours, mutations and changes in activity of TCA cycle enzymes have been detected including citrate synthase, aconitase (Schlichtholz et al. 2005; Singh et al. 2006), fumarate hydratase (Tomlinson et al. 2002), succinate dehydrogenase (Bardella et al. 2011) and isocitrate dehydrogenase (Balss et al. 2008). However, depending on the anatomical location of the tumour, some of these genes can be found to be either upregulated or downregulated, and induce different oncogenic processes. Mutations of isocitrate dehydrogenase for example are frequently found in brain tumours (Balss et al. 2008), and are suggested to raise 2-hydroxyglutarate levels (Dang et al. 2009), to help drive oncogenic promoting processes through DNA hypermethylation (Figueroa et al. 2010) and histone modification (Lu et al. 2012). Moreover, in cervical carcinoma cell lines mutations in citrate synthase are reported to cause downregulation in support of a glycolytic switch (Lin et al. 2012). On the other hand upregulation of citrate in pancreatic cancer has been hypothesised to favour synthesis of fatty acids (Schlichtholz et al. 2005), which fuels pancreatic tumour progression (Blum and Kloog 2014; Witkiewicz et al. 2008). The heterogeneity between tumour types and requirements is therefore extremely variable, and a generic role for these enzymes in cancer is somewhat debatable.

### mtDNA mutations in cancer cells

The detection of somatic mutations in the mtDNA of tumours has been documented in a range of cancers, such as colon, breast, liver and lung cancers (Chatterjee et al. 2006). Moreover the importance of mtDNA mutations in the metabolic rewiring and functioning of cancer cells is amplified by a loss of tumourigenicity by brain and breast cancer cells after the depletion of mtDNA (Cavalli et al. 1997; Weinberg et al. 2010). Comprising most protein subunits and the largest of all respiratory chain complexes, mtDNA mutations in complex I are the most commonly reported in tumours (reviewed by Gaude and Frezza 2014). Mutations in complex I mtDNA genes have been suggested to be required for a glycolytic switch (Calabrese et al. 2013) and ROS-driven metastasis (He et al. 2013; Ishikawa et al. 2008) by cancer cells. However, there appears to be a diverse tumorigenic potential of different mtDNA mutations affecting complex I activity with severe deficiency reducing tumorigenesis in osteosarcoma cells in vitro and in vivo (Iommarini et al. 2014). Mutations in mtDNA encoding genes of complexes III (Dasgupta et al. 2009), IV and V are also thought to influence tumourigenesis. Mutations in Complex III are reported to increase cancer cell growth through an increase in ROS production and apoptotic resistance (Dasgupta et al. 2009).

There are contrasting reports on the role of mtDNA and nuclear DNA encoded complex IV subunits in cancer progression. An upregulation of nuclear encoded subunits of complex IV have been detected in leukaemia cells which increases cellular OXPHOS and exacerbates ROS production (Chen and Pervaiz 2009), whereas mutations of mtDNA encoded complex IV subunits which decrease OXPHOS and increase ROS production have been detected in prostate cancer and ovarian tumours (Petros et al. 2005). This suggests a bias towards oncogenic promotion by nuclear encoded complex IV subunits, with mtDNA encoded complex IV subunit activity supressing tumour growth (Gaude and Frezza 2014).

Complex V is the final enzyme of the OXPHOS system and forms part of the permeability transition pore for calcium flux and apoptosis. Mutations in complex V have been suggested to cause apoptotic resistance, promoting cancer cell survival (Shidara et al. 2005). An elegant study by Gaude et al. engineered a cytoplasmic hybrid (cybrid) cell line with various heteroplasmy levels of the m.8993 T > G mutation in *MT*-*ATP6*, termed mTUNE lines. They showed that at high levels of heteroplasmy mTUNE cells made a glycolytic switch that was coupled by malate dehydrogenase 1 to reductive carboxylation of glutamine—a process that drives cell proliferation and migration. This switch was lost when the heteroplasmy level of the m.8993T > G mutations was low (Gaude et al. 2018). These data provide evidence that high-level heteroplasmic mtDNA mutations can promote an oncogenic metabolic phenotype. Other evidence suggesting that mtDNA mutations can promote a pro-oncogenic metabolic phenotype is the observed upregulation of de novo serine synthesis and one carbon metabolism that has been shown to occur in both cell lines with mtDNA mutations or depletion (Bao et al. 2016), and in animal models of mitochondrial myopathy (Khan et al. 2017; Nikkanen et al. 2016). Upregulation of these pathways has also been shown to promote cancer cell growth in animal models of intestinal cancer and lymphoma (Maddocks et al. 2017).

Alongside tumour growth and proliferation, the multi-step process of metastatic disease development also requires certain aspects of mitochondrial functioning. Metastasis first involves the epithelial to mesenchymal transition of cancer cells to allow migration (Nieto et al. 2016). The invasive potential of cancer cells during this transition is suggested to be supported and manipulated by mitochondrial biogenesis, OXPHOS and dynamics (Caino et al. 2016; LeBleu et al. 2014), as well as mitochondrial metabolites such as fumarate (Sciacovelli et al. 2016). Moreover, assays in mice showed high metastatic potential of cells with mutations in *MT*-*ND6* of complex I, linking metastasis with the overproduction of ROS (Ishikawa et al. 2008). Other studies have further identified metastatic dissemination to be promoted by mitochondrial ROS overproduction and associated signal transduction cascades (Comito et al. 2011; Porporato et al. 2014). Protection of cancer cells against ROS is partially due to increased glucose flux through the pentose phosphate pathway leading to antioxidant production, thus promoting their survival (Schafer et al. 2009). However, beyond a certain threshold, levels of ROS may inhibit metastasis (Piskounova et al. 2015). A recent study has shown that during metastasis, detachment of the tumour cells from the extracellular matrix followed by dissemination through the circulation is a rate limiting step in the process. This process is enhanced by reduced mitochondrial capacity and a reliance on glycolysis for ATP production which is supported by reductive carboxylation of glutamine; if this is prevented there is induction of ROS production and cell death, and inhibition of metastatic spread (Labuschagne et al. 2019).

## Conclusions

**Fig. 2:**
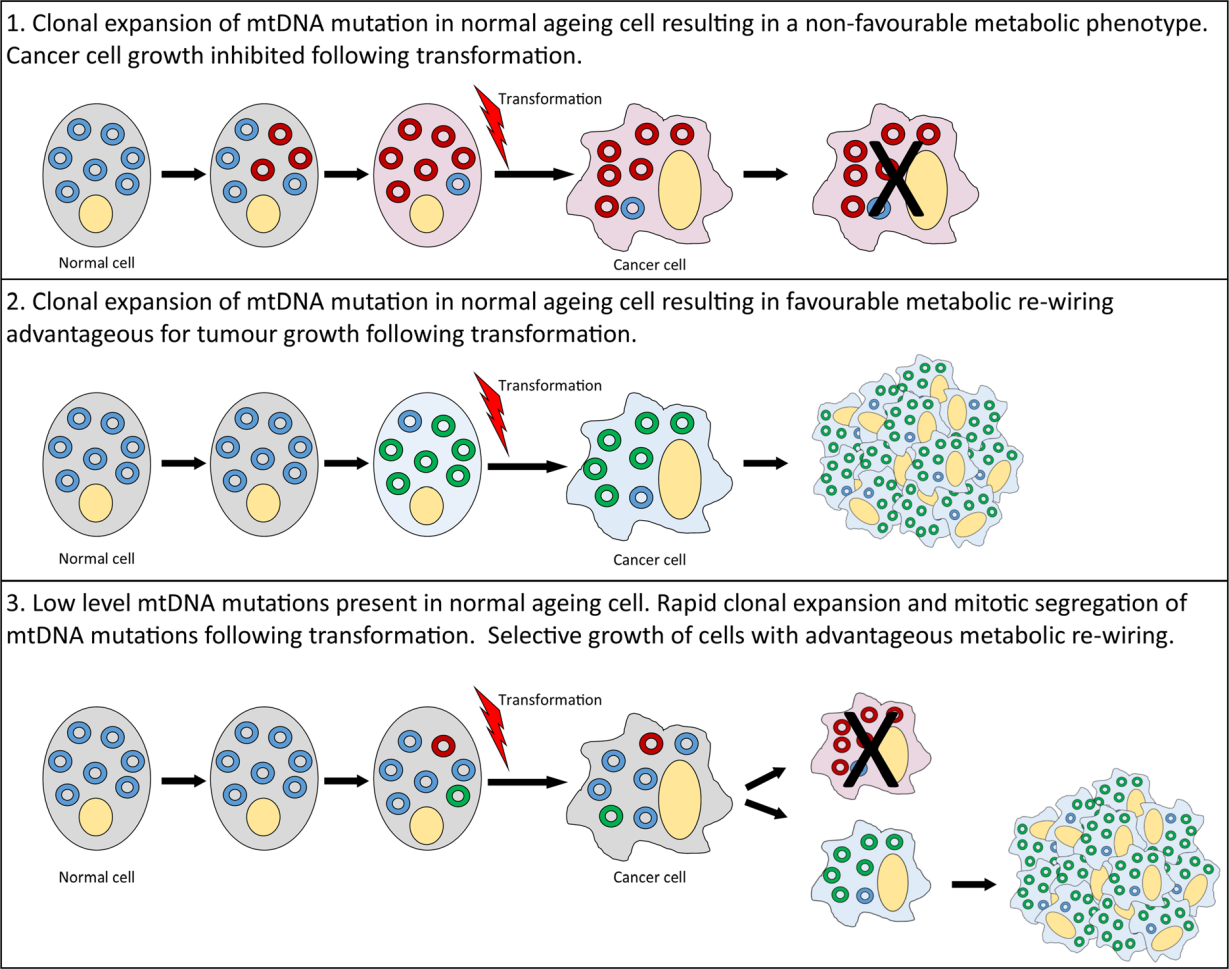
Schematic to show the hypothesised mechanisms by which mitochondrial DNA mutations may become manifest in tumours and either inhibit or promote tumour growth and/or progression. WT mtDNA is depicted in blue. Mutated mtDNA molecules conferring an advantageous metabolic phenotype for tumour growth are shown in green, and those causing a detrimental metabolic phenotype are shown in red

Metabolic rewiring is an important feature of many cancer types with metabolism moved away from OXPHOS towards anabolic pathways, providing biosynthetic building blocks for cell proliferation and migration. Mutations of the mtDNA are frequently observed in a number of solid tumour types (Ju et al. 2014; Salas et al. 2005) and are very common in normal ageing mitotic tissues. Importantly, non-cancer cells with OXPHOS defects due to mtDNA mutations upregulate some of the same metabolic pathways as those upregulated in cancers; posing an interesting question as to whether ageing cells with mtDNA mutations provide a favourable metabolic environment for tumour growth following transformation. Alternatively, as mtDNA mutations clonally expand rapidly in proliferating cells, this may occur during the tumour development, generating subsets of cell clones with a more favourable metabolic profile that out-compete other clones and promote tumour progression and metastasis (Fig. 2). Whether mtDNA mutations are drivers or passengers in the oncogenic process is still a matter of debate. It is likely that they will have differential effects depending on the tissue of origin and the specific mtDNA mutation present. In addition, further research sub-classifying tumours into those with and without mtDNA mutations and the effect of those mutations on tumour cell metabolism, may help to elucidate the functional effects of such mutations in the tumorigenic process, and whether mtDNA mutations cause metabolic vulnerabilities that may be exploited therapeutically.
